# 1094. Comparison of Demographic and Clinical Characteristics in Survivors Vs. Decedents with Multisystem Inflammatory Syndrome in Children (MIS-C) During the COVID-19 Pandemic—CDC National Surveillance, February 2020-December 2022

**DOI:** 10.1093/ofid/ofad500.067

**Published:** 2023-11-27

**Authors:** Anna R Yousaf, Katherine N Lindsey, Ami B Shah, Allison D Miller, Michael J Wu, Michael Melgar, Laura D Zambrano, Angela P Campbell

**Affiliations:** Centers for Disease Control and Prevention, Atlanta, GA; Centers for Disease Control and Prevention, Atlanta, GA; General Dynamics Information Technology, Atlanta, Georgia; Centers for Disease Control and Prevention, Atlanta, GA; Centers for Disease Control and Prevention, Atlanta, GA; Centers for Disease Control and Prevention, Atlanta, GA; Centers for Disease Control and Prevention, Atlanta, GA; CDC, Atlanta, GA

## Abstract

**Background:**

Multisystem inflammatory syndrome in children (MIS-C) is a rare but severe complication of SARS-CoV-2. Identifying clinical and demographic characteristics associated with MIS-C mortality and description of MIS-C decedents over time may help identify children at increased risk for poor outcomes and potentially inform treatment decisions.

**Methods:**

This investigation included all MIS-C cases with known outcome reported to CDC MIS-C national surveillance from February 2020─December 2022. Clinical and demographic characteristics were compared between survivors and decedents. Decedents were described over time and classified by date of MIS-C illness onset into five pandemic waves corresponding to five peaks of MIS-C activity (Figure 1): (1) 2/19/2020─6/28/2020, (2) 6/29/2020─10/17/2020, (3) 10/18/2020─7/8/2021, (4) 7/9/2021─12/7/ 2021, and (5) 12/8/2021─12/31/2022. Waves 4 and 5 were periods of predominant Delta and Omicron SARS-CoV-2 variant circulation, respectively.

**Figure 1.**
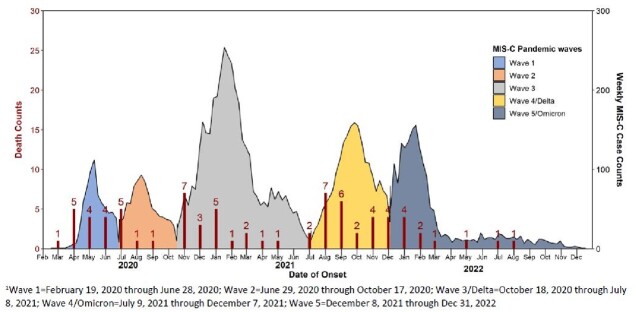
Multisystem Inflammatory Syndrome in Children (MIS-C) Decedents by Pandemic MIS-C Wave, February 19, 2020-December 31, 2022, N=76

**Results:**

Seventy-six decedents and 8,691 survivors were reported (Table). A higher proportion of decedents compared with survivors were aged 16-20 years (41% vs 9%; p< 0.01), from the South (55% vs 41%; p=0.01) and had MIS-C illness onset in wave 1 (18% vs 7%; p< .01). Half (51%) of decedents had an underlying medical condition compared with 24% of survivors (p< 0.01). A higher proportion of decedents than survivors had severe cardiovascular (95% vs 76%), respiratory (99% vs 36%), renal (59% vs 19%), and neurologic (30% vs 4%) involvement, and fewer had mucocutaneous (33% vs 75%). Deaths in the 16-20 age group were predominant in waves 1-4 but comprised only 15% of deaths in wave 5, when 57% of decedents were aged 0-4 years (Figure 2). Race and ethnicity of decedents varied over time. Severe cardiovascular and respiratory involvement was high ( >85-100%) over time in decedents; other organ manifestations varied by MIS-C wave.

**Table.**
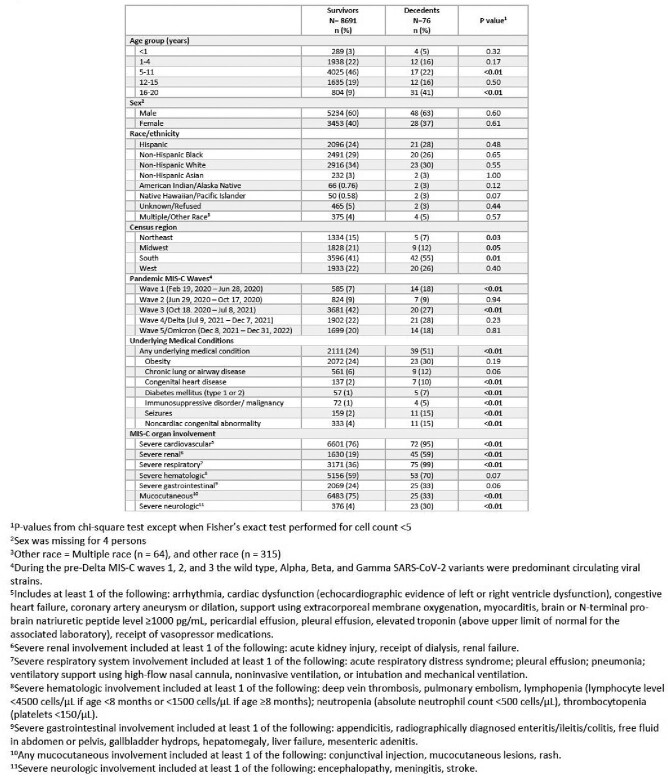
Demographic and Clinical Characteristics of Children with Multisystem Inflammatory Syndrome in Children (MIS-C)-CDC National Surveillance, February 2020-December 2022

**Figure 2.**
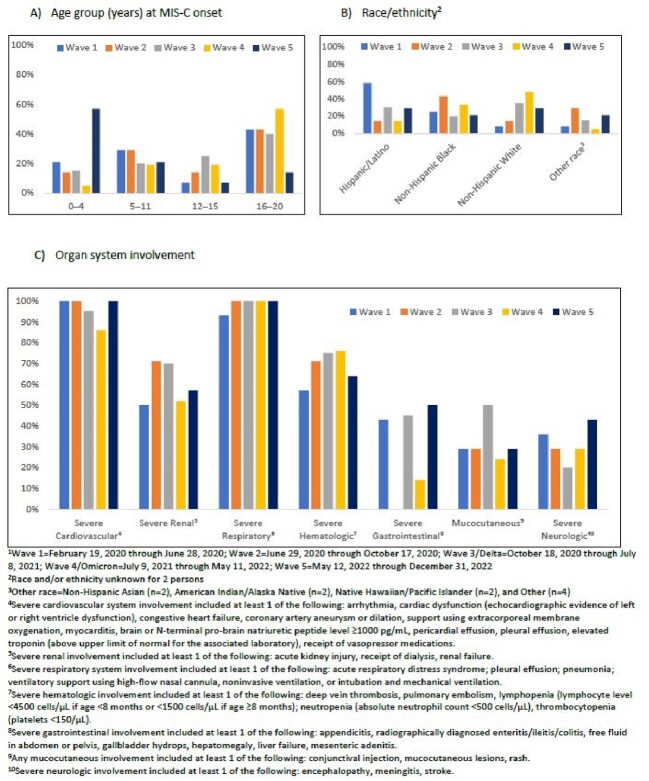
Demographic Characteristics and Organ Involvement of Children with Multisystem Inflammatory Syndrome in Children (MIS-C) Who Died Stratified by Pandemic MIS-C Wave, February 19, 2020-December 31, 2022, N=76

**Conclusion:**

Older age (16-20 years), residing in the South, illness early in the pandemic, underlying medical conditions, and severe organ involvement were all significantly associated with death among children with MIS-C. MIS-C decedent characteristics are dynamic and may continue to evolve with changes in underlying population immunity, SARS-CoV-2 transmission, and variant emergence.

**Disclosures:**

**All Authors**: No reported disclosures

